# Supplementary material for: Ecology and Infection Status of Sand Flies in Rural and Urban Cutaneous Leishmaniasis Endemic Areas in Northwest Ethiopia
Source: Trop Med Infect Dis. 2024 Feb 23;9(3):52. doi: 10.3390/tropicalmed9030052 (PMC10974852; doi:10.3390/tropicalmed9030052)
Supplement: Supplementary file 1 [file tropicalmed-09-00052-s001.zip › tropicalmed-2767040-supplementary.pdf]

Supplementary Materials:

Table S1. Proportions of engorgement status of female *Phlebotomus longipes* sand flies, in urban Addis-Alem and rural Gindmeteaye.

Engorgement status	Addis-Alem (urban)	Gindmeteaye (rural)	Combined
Blood fed	31 (13%)	57 (28%)	88 (20%)
Gravid	22 (10%)	37 (18%)	59 (14%)
Non-fed/sugar-fed	175 (77%)	108 (54%)	283 (66%)
**Total**	**228**	**202**	**430**
